# Dwarfs on the Shoulders of Giants: Bayesian Analysis With Informative Priors in Elite Sports Research and Decision Making

**DOI:** 10.3389/fspor.2022.793603

**Published:** 2022-03-17

**Authors:** Anne Hecksteden, Sabrina Forster, Florian Egger, Felix Buder, Ralf Kellner, Tim Meyer

**Affiliations:** ^1^Institute of Sports and Preventive Medicine, Saarland University, Saarbrücken, Germany; ^2^Quantitative Methods and Statistics, Saarland University, Saarbrücken, Germany; ^3^Financial Data Analytics, University of Passau, Passau, Germany

**Keywords:** Bayesian statistics, individual response, cold-water immersion, methodology, replicate crossover

## Abstract

While sample sizes in elite sports are necessarily small, so are the effects that may be relevant. This conundrum is complicated by an understandable reluctance of athletes to comply with extensive study requirements. In Bayesian analyses, pre-existing knowledge (e.g., from sub-elite trials) can be formally included to supplement scarce data. Moreover, some design specifics for small sample research extend to the extreme case of a single subject. This provides the basis for actionable feedback (e.g., about individual responses) thereby incentivising participation. As a proof-of-concept, we conducted a replicated cross-over trial on the effect of cold-water immersion (CWI) on sprint performance recovery in soccer players. Times for 30 m linear sprint and the initial 5 m section, respectively, were measured by light gates before and 24 h after induction of fatigue. Data were analysed by Bayesian and by standard frequentist methods. Informative priors are based on a published metaanalysis. Seven players completed the trial. Sprint performance was 4.156 ± 0.193 s for 30 m linear sprint and 0.978 ± 0.064 s for the initial 5 m section. CWI improved recovery of sprint time for the initial 5 m section (difference to control: −0.060 ± 0.060 s, *p* = 0.004) but not for the full 30 m sprint (0.002 ± 0.115 s, *p* = 0.959), with general agreement between Bayesian and frequentist interval estimates. On the individual level, relevant differences between analytical approaches were present for most players. Changes in the two performance measures are correlated (*p* = 0.009) with a fairly good reproducibility of individual response patterns. Bayesian analyses with informative priors may be a practicable and meaningful option particularly for very small samples and when the analytical aim is decision making (use / don't use in the specific setting) rather than generalizable inference.

## Introduction

Research in elite sports is associated with a characteristic set of challenges. Most obviously this concerns the specific setting e.g., limited access to athletes and their understandably critical view on (study) requirements that are not exclusively intended to support individual performance. Beyond those gates, the main scientific challenge consists of finding a solution to the „small *n*—small effects conundrum.” In other words: While the number of potential participants is necessarily small, so are the effects that may be relevant in this highly competitive environment. This constellation conflicts with the general rule that small effects (or highly precise estimates) require large sample sizes. Importantly, this consideration extrapolates to the extreme when assessing and counselling individual athletes (*n* = 1).

There are many tweaks for optimising insights that may be gained from small studies. A comprehensive discussion of dealing with small sample sizes in an elite sports context has been recently published elsewhere (Hecksteden et al., 2021). Two particularly promising options are study designs that include some form of replication on the individual level (e.g., a replicated cross-over design) (Hilgers et al., 2018; Hecksteden et al., 2021) and a switch from mainstream frequentist to Bayesian statistics (Greenland, 2006; Greenland et al., 2016; Van de Schoot, 2020). Collecting several observations per individual reduces the number of study participants needed to get enough datapoints for group-based analyses (Hilgers et al., 2018; Hecksteden et al., 2021). Moreover, replication on the level of the individual enables valid assessment of individual responses (Senn et al., 2011; Hecksteden et al., 2015), which is what is called for in elite sports decision making (“Shall this particular athlete apply the intervention?”). Bayesian statistics offer several advantages for small trials, most prominently the possibility to formally include pre-existing knowledge about the effect of interest (e.g., the efficacy of an intervention). This “informative prior” may be derived from a variety of sources e.g., from larger trials in sub-elite athletes, meta-analyses or in some cases from routine data (Hecksteden et al., 2017) or expert judgement (Van de Schoot, 2020). As a result, data analysis for a given trial (or one specific athlete) (Sottas et al., 2007; Hecksteden et al., 2017; Barth et al., 2019) does not have to start from zero. Rather, the current data are used to update the prior which already indicates an expectable range and thereby augments the small elite trial. Of note, Bayesian methods can also be employed when no adequate prior knowledge is available or when analyses are intentionally limited to the current dataset. The “uninformative” (“flat”, “diffuse”) priors used in these situations sidestep the need to derive and justify a specific informative prior. However, if presumably valid prior knowledge is available, informative priors may give studies with (very) small sample sizes a decisive head-start. A second relevant advantage concerns the interpretation of interval estimates: Bayesian credible intervals indicate the range within which the parameter of interest (e.g., representing the efficacy of an intervention) is located with a certain probability. The frequentist counterparts, confidence intervals, are often interpreted that way, but their true meaning is less straight forward (“If the experiment was repeated many times, a proportion equal to the confidence level would include the true value”). Finally, the Bayesian notion of probability as a subjective believe contingent on the current state of knowledge is less dependent on many datapoints as compared to the frequentist “long run relative frequency.” Although this does not eliminate the advantages of a high number of observations, Bayesian analyses are generally less dependent on sample size (Van de Schoot, 2020). Box 1 summarises relevant advantages of Bayesian analyses with informative priors in elite sports.

Box 1 **Potentially relevant advantages of Bayesian analyses with informative priors in elite sports.**

- “Head-start” by formally including pre-existing knowledge about the parameter of interest (e.g., from trials in sub-elite athletes)
- Provides information crucial to gauge practical relevance (credible interval)
- Lower dependence on sample size (philosophically and in the applied setting)
- Extends to the assessment of individual athletes (*n* = 1) (Individual results may serve as incentive for athlete participation).

It is understood that Bayesian analyses are also beset with relevant drawbacks. From the perspective of the applied scientist two particularly relevant: (1) Computational challenges (“How to do it?”). (2) The risk of bias due to an unavoidable degree of subjectivity in the prior distributions even when based on previous scientific work or other empirical evidence (e.g., routine data). Finally, it should be noted that Bayesian and frequentist approaches to statistics are not (or at least do not have to be regarded as) mutually exclusive (Senn, 2011). Rather, they may be most suitable for different analytical aims (Senn, 2011). An illustrative example is the combination of inference about the efficacy of an intervention with feedback to participating athletes about their individual responses intended to inform decision making (use/don't use). The current work has been undertaken as a proof-of-principle for combining replication on the individual level and Bayesian analyses with informative priors in the context of high-performance sport.

Aiming at a practically relevant empirical example, this trial investigates the efficacy of cold-water immersion (CWI) for enhancing recovery of sprint performance in soccer players on the group- as well as on the individual level. In a nutshell, improving recovery enables athletes to sustain higher training loads without accumulating recovery deficits and / or to restore performance capacity faster in competitive situations. Therefore, considering the high training loads and tiny differences in performance in current high-performance sports, recovery interventions are highly relevant. A concise overview is provided in Kellmann et al. (2018). From the multitude of proposed recovery interventions, CWI is among the few with substantial evidence in favour of a beneficial (main) effect on performance recovery in athletes (Poppendieck et al., 2013; Kellmann et al., 2018). However, effect sizes are still small (Poppendieck et al., 2013; Kellmann et al., 2018). Moreover, efficacy of CWI depends on several factors including the dimension of physical capacity in question (endurance, strength, speed etc.), the characteristics of the physical load used to induce fatigue and the timeframe (Poppendieck et al., 2013). Taken together, CWI seems to have the potential for beneficial effects of relevant magnitude in high-performance sports—but negligible or even adverse effects cannot be ruled out. Therefore, it seems reasonable to verify the prior expectation of a beneficial effect of CWI for the particular framework conditions and athletes (Kellmann et al., 2018). From a methodological perspective, the effects of CWI are fast (with pre to post-test timeframes between hours and days) and reversible, a combination which fulfils the requirements for a replicate cross-over design (Hecksteden et al., 2021). The choice of dependent variables equally followed the rationale of sport-specific, practical relevance. Therefore, changes in recovery status were measured as changes in discipline related performance (Hecksteden et al., 2016).

## Materials and Methods

### General Design and Data Collection

The efficacy of whole-body CWI was investigated in soccer players (German 4th league) using a replicated cross-over design. A standardised, intensive, soccer-specific exercise bout comprising small-sided games and running drills was used to induce fatigue. In intervention periods, whole-body CWI was applied within 30 min after the end of exercise. Duration of CWI was 15 min and water temperature was 12–15°C as recommended for optimal efficacy (Poppendieck et al., 2013). Passive recovery (seated rest for 15 min) was employed as control condition. Changes in recovery status were measured as changes in discipline related performance (Hecksteden et al., 2016) (30 m linear sprint time and the time for the initial 5 m section thereof; means of 3 trials, light gate timing, self-timed start from 1 m behind the first light gate). Post-tests were conducted 24 h after the pre-test. Wash-out between testing periods was 1 week. All participants without missing data were analysed (*n* = 7). The protocol was approved by the local ethics committee (Ärztekammer des Saarlandes, approval number 176/18).

### Prior

Prior distributional parameters for the efficacy of whole-body CWI (β_CWI_) are based on the metaanalysis published by Poppendieck et al. (2013) the rationale being to characterise the range of plausible values pre-trial (Wagenmakers et al., 2016; Hecksteden et al., 2017; Van de Schoot, 2020). A detailed account is provided as a Supplementary File.

In short, based on the meta-analysis a beneficial effect of CWI around 5% of initial performance may be expected. An adverse effect of CWI seems unlikely, which may translate to the expected value being 2 standard deviations away from zero. Considering baseline performance of our study participants, the following informative priors for β_CWI_ were used: N (0.049, 0.024) (a normal distribution (“N”) with mean 0.049 and standard deviation 0.024) for the initial 5 m section and N (0.208, 0.104) for 30 m sprint time. For comparison, group-based analyses were also conducted with a flat (improper) prior for β_CWI_. Priors for intercept and residual variance were N (0, 100) and uniform (0, 1000), respectively.

### Data Analysis

All analyses were conducted in R [version 3.6.3^1^) and Stan (version v2.25.0 (Team, 2020a)]. The main specific packages used were lme4 (Bates et al., 2015), lmerTest (Kuznetsova et al., 2017), and RStan (Team, 2020b).

Analyses within the frequentist and Bayesian frameworks, respectively, were based on similar mixed effects models (outcome: pre-post difference in sprint time, fixed effect: condition (CWI vs. control), random effects: ID and condition-by-ID interaction. Of note: A model based on measured values would be preferable in principle, however such an approach would increase complexity considerably and has therefore not been implemented. Interval estimates (credible or confidence intervals, respectively) were calculated as main results.

Raw data, statistical code (R Markdown file and required supporting stan and rds files) are made available in the following public repository: https://doi.org/10.5281/zenodo.5819654. Raw data can also be accessed as a supplement to this work.

#### Standard (Frequentist) Approach

Desciptive statistics are given as means ± standard deviation if not otherwise indicated. A linear mixed model with random intercept and slope was used to analyse differences between conditions. The lme4 and lmerTest packages were used for mixed modelling. The significance level was set at α <0.05.

#### Bayesian Approach

Bayesian analyses were conducted using Markov chain Monte Carlo (MCMC) simulation implemented in R and Stan. Posterior distributions for the difference between conditions were estimated based on a linear mixed model with random intercept and slope (cp. Supplementary Stan Files). Simulations were based on 5000 iterations. Credible intervals are 95% highest posterior density intervals (HPDI).

#### Analyses on the Individual Level

On the individual level (frequentist), confidence intervals for the difference between conditions were calculated by fitting a linear model for each subject (dependent variable: pre-post difference, predictor variable: recovery condition). This approach based exclusively on datapoints from the concerned individual. For comparison, **Figure 4** also displays intervals based on the individual mean for the difference between conditions and the (group-based) standard error for the fixed effect in the mixed model (Bayesian) credible intervals (HPDI) are simulation based in analogy to the group-based results.

## Results

### Frequentist Results

**Figure 1:**
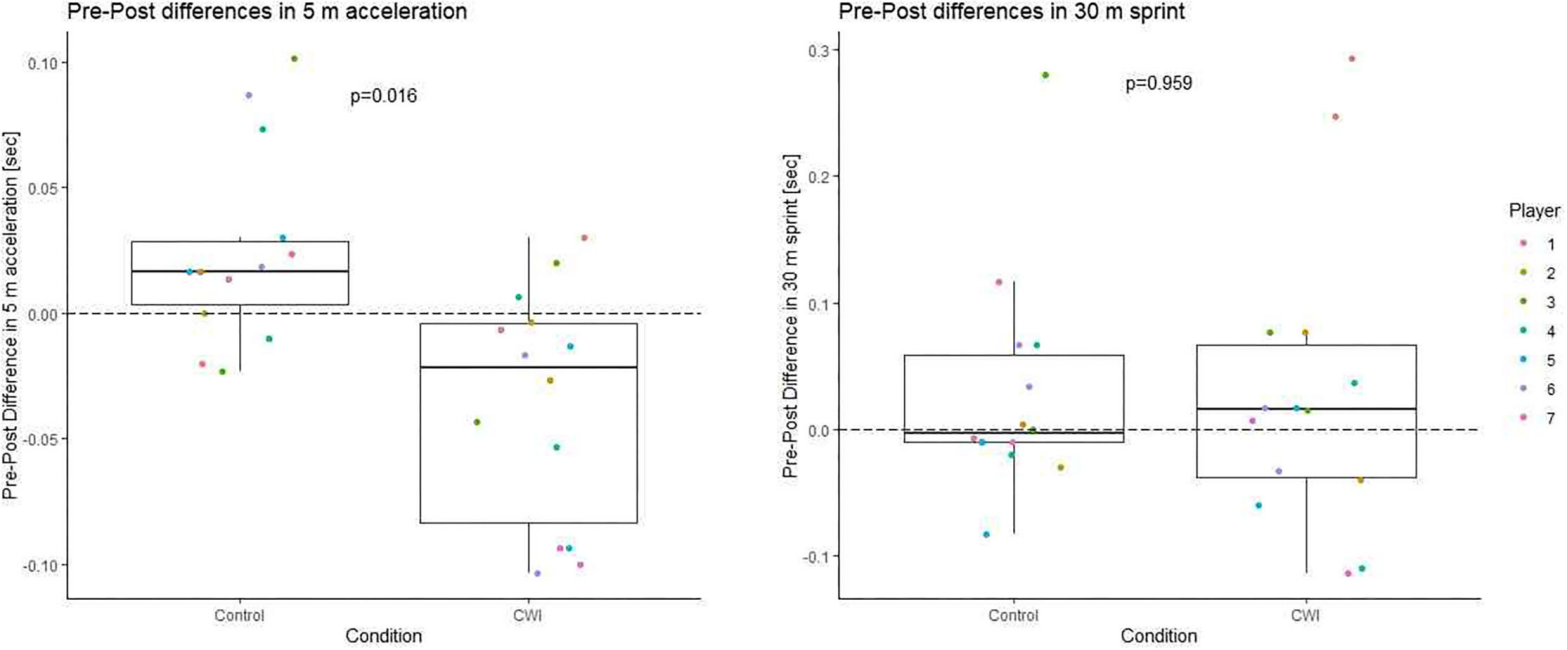
Boxplot of pre-post changes in sprint time for 30 m sprint and the initial 5 m section depending on the recovery condition p-values are derived from the linear mixed model described in the methods section.

Observed sprint performances follow expectations for players in the highest German amateur soccer league (initial 5 m: 0.978 ± 0.064 s, 30 m linear sprint: 4.156 ± 0.193 s). Pre-post changes are displayed in Figure 1. The difference in pre-post changes between CWI and control was −0.060 ± 0.060 s for the initial 5 m section and 0.002 ± 0.115 s for 30 m sprint. Confidence intervals are included in **Figure 3**. The difference between conditions reached statistical significance for the initial 5 m section but not for the whole 30 m sprint distance.

**Figure 2:**
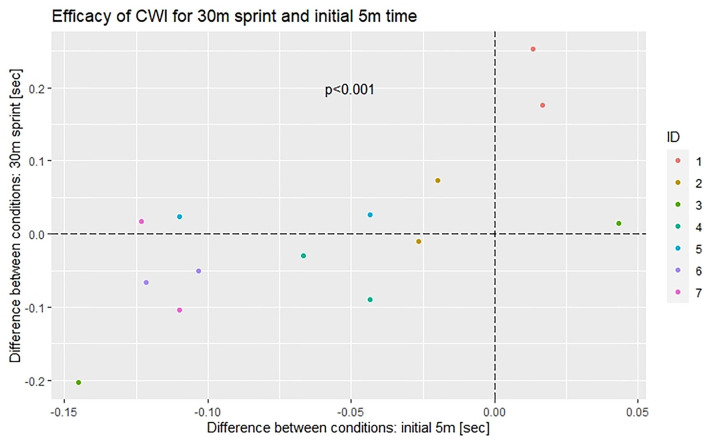
Difference between conditions for 30 m sprint and initial 5 m section. Dashed lines highlight zero difference between conditions.

Differences in pre-post changes between conditions for the two distances are correlated (Figure 2) with a fairly good reproducibility of response patterns on the individual level across the 2-fold replicated cross-over (**Figure 4**). Salient is subject #1 with a consistent adverse response across distances and repetitions.

### Bayesian Results

**Figure 3:**
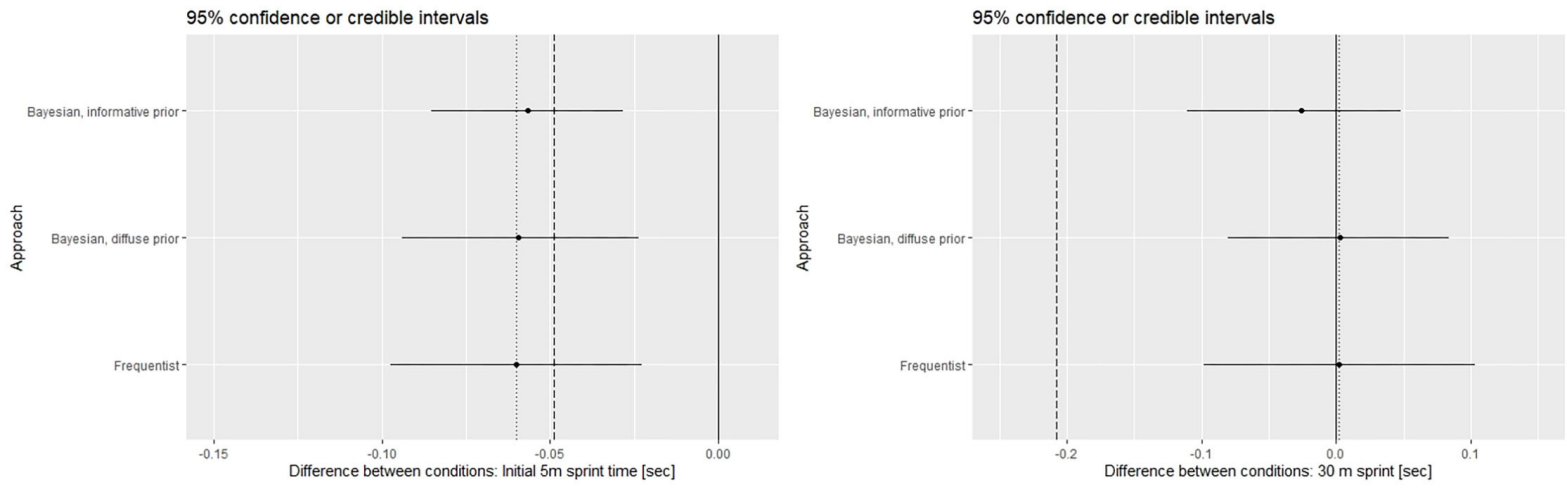
Efficacy of cold-water immersion. 95% Bayesian credible intervals (highest posterior density intervals) and 95% frequentist confidence intervals, respectively. Dashed vertical line: prior mean, Dotted vertical line: data mean, Solid vertical line: Zero Bayesian credible intervals and frequentist confidence intervals calculated with the same data are displayed in one figure for comparison. Please keep in mind their fundamental disparity.

Figure 3 displays differences between CWI and control. Bayesian credible intervals as calculated with informative and diffuse priors, respectively, are displayed in comparison to the frequentist confidence interval. To guide interpretation, prior and data mean as well as zero are indicated. The left panels illustrate results for the initial 5 m section, the right panels for 30 m sprint.

Evidently, the effect of CWI is even slightly larger than expected for 5m acceleration but negligible (and thereby considerably smaller than expected) for 30 m sprint time.

With respect to the comparison between analytical approaches several aspects may be noted, which all comply with expectations: (i) Frequentist confidence intervals and Bayesian credible intervals as calculated with the diffuse prior are naturally centred on the data mean. (ii) Location of the credible interval as calculated with the informative prior is a compromise between prior and data means. (iii) Interval width tends to be smallest for the credible interval as calculated with informative prior. (iv) Bayesian credible intervals are calculated as highest posterior density intervals (HDPI) and therefore not necessarily symmetrical. However, deviations from point symmetry are generally minor.

### Results on the Individual Level

**Figure 4:**
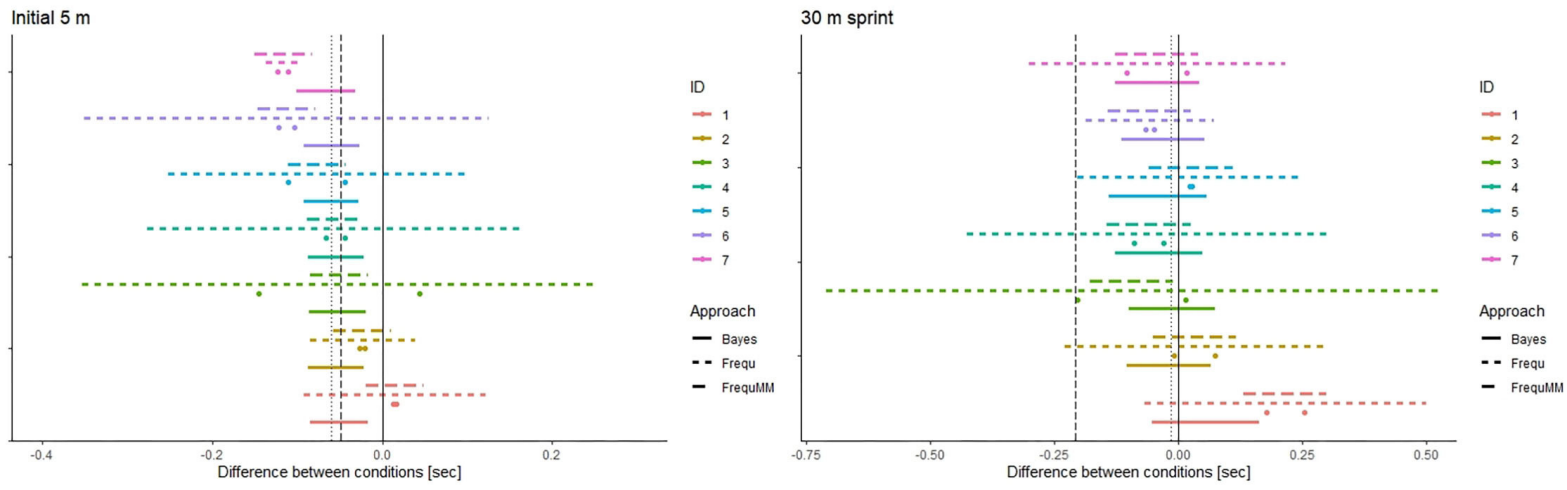
Efficacy of cold-water immersion on the individual level. 95% Bayesian credible intervals (highest posterior density intervals, Approach = Bayes) and 95% frequentist confidence intervals (Approach = Frequ), respectively. Intervals calculated from individual means and the (group-based) standard error for the fixed effect (Approach = FrequMM) are displayed for comparison. Points are observed differences in pre-post changes between conditions. HPDI have been calculated with informative priors. Subjects are ordered by posterior β_CWI_ for 5 m acceleration. Dashed vertical line: prior mean, Dotted vertical line: data mean, Solid vertical line: Zero Bayesian credible intervals and frequentist confidence intervals calculated with the same data are displayed in one figure for comparison. Please keep in mind their fundamental disparity.

Figure 4 illustrates confidence and credible intervals on the individual level together with the observed differences in pre-post changes between conditions.

As already apparent in Figures 1, 2, 4 illustrates considerable interindividual variation in the effect of CWI on sprint performance recovery.

Regarding the comparison between frequentist and Bayesian intervals, the difference between approaches is more pronounced compared to the group-based results in Figure 3. While frequentist confidence intervals naturally remain centred on the data mean, Bayesian credible intervals are influenced by the prior mean. The width of credible intervals is less dependent on the variability in observed individual values as compared to confidence intervals based exclusively on individual datapoints (where this dependence is most pronounced given the tiny n). Of note, using the (group-based) standard error of the fixed effect together with the individual mean may be an option to circumvent this issue in a frequentist framework.

From the applied perspective of providing feedback and recommendations to individual players, the difference between approaches seem relevant for 6 out of 7 individuals. Except for subject 7 (most pronounced mean difference between conditions, little variation between cross-over 1 and 2) all frequentist confidence intervals span the zero line. By contrast, Bayesian credible intervals for the initial 5 m section time are all in the negative range indicating a beneficial effect of CWI.

## Discussion

With uninformative priors, Bayesian and frequentist results were in general numerical agreement indicating a beneficial effect of CWI on performance recovery for the initial 5 m section but not for 30 m sprint (Figure 3). Of note, the pre-post difference in 30 m sprint time with the control condition is already negligibly small (Figure 1), leaving little room for improvement by CWI. The reduction in neuromuscular function and explosiveness associated with exercise induced fatigue [specifically with the intermittent, intensive exercise bouts in team sports (Wiewelhove et al., 2015)] together with the typical (not near-maximal) load used to induce fatigue could plausibly explain impaired acceleration over the initial section of the 30 m sprint only. However, this remains speculative at this point and replicability of the difference remains to be verified.

The informative priors caused only minor changes in credible intervals as compared to uninformative priors even with our relatively small dataset and despite the difference in prior and data means (Figure 3, upper panels). All other things being equal, the impact of the prior is dependent on the number of datapoints. This n-dependent weighting between prior and data extends to the individual level (Figure 4) and reflects the robustness against variation (random or not) provided by the “rest mass” of the informative prior. However, with the fairly good reproducibility of individual responses in our data, this comes at the cost of identifying truly extreme cases (seemingly subject #1) only with several datapoints able to “override” the prior. Sensitivity analyses (conducting the analysis with a range of prior distributions) can be used to verify the relevance of plausible variations in the prior. However, this comes at the cost of added complexity in analysis and communication. We therefore did not conduct sensitivity analyses in this work which mainly aims to probe and illustrate the basic concept.

The above results underline that Bayesian updating of an empirically justified informative prior provides a traceable summary of the evidence available at a specific timepoint–but is not guaranteed to provide more accurate estimates under any circumstances. Ex ante it is impossible to tell with certainty if considering a specific, empirically justified prior will be beneficial in terms of predicting future instances of e.g., response to CWI in similar or the same athletes. However, it may be argued that if there is sufficient evidence in support of a specific intervention to be tested in elite athletes, this “range of expectable values” may also merit to be considered as part of a new state of knowledge after data have been collected. In other words, Bayesian analysis with informative priors is particularly appropriate if the analytical aim is on decision making rather than on generalizable inference (Senn, 2011), available data on the level of interest is scarce (Sottas et al., 2007; Van de Schoot, 2020), and supposedly applicable prior knowledge is available (Aitken and Taroni, 2004; Hecksteden et al., 2017). As already pointed out in the introduction, this situation regularly occurs in elite sports.

If transferability of previous empirical results seems limited, this additional uncertainty can be considered by increasing the spread of the prior distribution and thereby decreasing its weight relative to the data (Van de Schoot, 2020). Performance outcomes may be especially prone to limited transferability of previous results because practically meaningful (valid) tests are frequently sport-specific and may differ between performance levels. Performance level by itself and sports discipline are other factors to be considered. In this work, we opted not to consider presumed transferability to avoid an additional degree of subjectivity in the prior.

The above considerations apply particularly for analyses on the individual level where the number of observations is lowest. Factors deciding the accuracy of an individual's posterior mean include the relation of the individual's mean response to the prior mean, as well as variation between and within individuals. From the perspective of providing feedback to the participating athlete it is important to note that these factors are unknown at the timepoint of testing and feedback. In observational settings with high numbers of individual measurements per subject, similar approaches have been shown to improve accuracy in sport-related contexts (Sottas et al., 2007; Hecksteden et al., 2017; Barth et al., 2019). This may be tentatively extrapolated to individual responses where it is difficult to obtain substantially more than 2 repetitions. On the part of data collection, assessing the “individual response” of an athlete to a specific intervention should be based on more than one application and the respective control period because otherwise variation on the level of interest remains unknown (Senn et al., 2011; Hecksteden et al., 2015). Therefore, if actionable feedback is to be given on the individual level, a replicated design seems mandatory (Senn, 2004; Hecksteden et al., 2015). Taken together (and in agreement with common sense) this approach can be described as a “scientifically guided try-out.”

While decision making within the scenario investigated is highly relevant in elite sports, the prevailing inferential perspective (“What can be concluded from the current data?”) remains valid. Bayesian methods with uninformative priors are a promising option for this complementary analytical aim particularly if the sample size is small (Van de Schoot, 2020). In this work, numerical limits of credible intervals from Bayesian analyses with uninformative priors and frequentist confidence intervals did not differ substantially. The more intuitive interpretation favours the Bayesian credible interval. Moreover, the posterior distribution (but not the frequentist confidence interval) allows to specify the probability of specific effect magnitude (Sainani, 2018). However, it is beyond the scope of this work to generally weigh the respective assets and drawbacks of the two approaches against each other. Importantly, the Bayesian and frequentist notions of probability and the respective analytical approaches are not mutually exclusive but can be used depending on analytical aim and framework conditions (Senn, 2011). Of course, the analytical strategy should be defined in advance.

Of note: This work does not aim to systematically expose—let alone evaluate—the principles, options, and previous applications in sports of Bayesian statistics. We rather aim to discuss the core approach and provide a worked example. To ensure transparency, reproducible code is provided and can be executed via the R Markdown file (https://doi.org/10.5281/zenodo.5819654) without previous experience in Bayesian methods or coding skill. By contrast, the code is not intended for direct productive operation. For a concise, non-technical introduction to Bayesian data analysis interested readers are referred to Wagenmakers et al. (2016) and Van de Schoot (2020). An excellent tutorial textbook has been published by Kruschke (2015). For an accessible introduction to mixed modelling we recommend (Brown, 2021).

## Conclusions

- Bayesian updating of an informative prior is a meaningful and practicable option for data analysis in elite sports particularly when the analytical aim is on decision making (e.g., use/don't use an intervention in the specific setting investigated) rather than generalizable inference.
- Using informative priors is particularly meaningful with very small samples and individual athletes. When combined with appropriate study designs actionable results may serve as important incentives for participation in scientific studies.
- The prior distribution for the parameter of interest denotes the “expectable range of values.” The prior should be traceably based on the available evidence pre-trial. Supposed limitations of transferability may be considered by increasing the spread of the prior, thereby decreasing it's impact on the posterior.

## Data Availability Statement

The original contributions presented in the study are included in the article/Supplementary Material, further inquiries can be directed to the corresponding authors.

## Ethics Statement

The studies involving human participants were reviewed and approved by Ärztekammer des Saarlandes, approval number 176/18. The patients/participants provided their written informed consent to participate in this study.

## Author Contributions

SF, FE, FB, RK, and TM have made substantial contributions to data collection, data analysis and interpretations as well as drafting and critical revision of the manuscript. All authors have approved the final version of the manuscript.

## Funding

The present study was funded by the German Institute of Sport Science within the project Exact – Expertise on Dealing with Small Sample Sizes in Elite Sports Research (ZMVI4-080511/19-20). Data collection was realized within the project REGman - Management of Regeneration in Elite Sports (IIA1-081901/12-16 and 17-20).

## Acknowledgements

The authors wish to thank Stephen Senn for expert statistical comments which enabled important improvements of the manuscript, Lars Donath for insightful feedback from the perspective of sports science, as well as Alexander Ferrauti, Mark Pfeiffer, and Michael Kellmann for their support of data collection.

## Conflict of Interest

The authors declare that the research was conducted in the absence of any commercial or financial relationships that could be construed as a potential conflict of interest.

## Publisher's Note

All claims expressed in this article are solely those of the authors and do not necessarily represent those of their affiliated organizations, or those of the publisher, the editors and the reviewers. Any product that may be evaluated in this article, or claim that may be made by its manufacturer, is not guaranteed or endorsed by the publisher.

## Footnotes

^1^Team (2020a). R 3.6.3.
