# Supplementary material for: Dwarfs on the Shoulders of Giants: Bayesian Analysis With Informative Priors in Elite Sports Research and Decision Making
Source: Front Sports Act Living. 2022 Mar 17;4:793603. doi: 10.3389/fspor.2022.793603 (PMC8970347; doi:10.3389/fspor.2022.793603)
Supplement: Supplementary file 1 [file Presentation_1.PDF]

Supplement: Specification of prior distributions

General aspects

The prior for the parameter of interest characterizes the range of plausible values for the difference in relative performance changes between CWI and the control condition (β_{CWI}).[1] Here, this pre-trial expectation for the efficacy of CWI has been based on the meta-analysis published by Poppendieck et al..[2] Of note, this rationale parallels the expert reasoning used to justify a trial in elite athletes in the first place.

In a second step, the “range of plausible values” is translated into distributional terms. Importantly, this step requires specifying distribution type. This seemingly theoretical aspect has large impact on the resulting credible intervals and thereby potentially on conclusions and practical recommendations. In this work, selection of distribution type has been informed by previous own data ([3] and denonymized data from routine assessment of soccer teams) as well as by theoretical and computational considerations. More detail is provided below.

While this proceeding is a basic way to specify an informative prior distribution for the effect of interest, it may be argued that the resulting prior is coherent in the sense that it can be (or even has been) used as basis for a bet on trial outcome [4]. One noteworthy limitation should be kept in mind: Although grounding the prior on empirical results is intuitive, observed distributions never represent the true value of a parameter in isolation but confounded with (at least) random variation. Therefore, spread will most likely be overestimated and distribution form for the parameter of interest may be obscured. Moreover, this phenomenon may plausibly lead to an overestimation of the true value e.g. for β_{CWI}.[5]

Taken together this way of specifying the prior may be a reasonable starting point. A comprehensive expose and discussion of the various options to characterize and implement prior distributions is required if Bayesian methods are to be established as part of the statistical toolbox in sports science and medicine.

Steps taken in this work

Distribution type

Distribution type for sprint related performances and their fatigue related changes (irrespective of CWI) was assessed using previous own research [3] and routine data. Distributions of raw values were generally symmetrical and compatible with a normal

distribution. For fatigue related changes a slight positive skewness became apparent (with performance decrements (increase in sprint time) being represented as positive values). However, assuming normality still seemed warranted considering computational advantages, the absence of conclusive theoretical arguments for another distribution form and the presumable dependence of this aspect on the specific group of subjects. Normality is also assumed for the intercept and a uniform distribution for the residual variance.

Distributional parameters

Prior distributional parameters for the efficacy of whole-body CWI (β_{CWI}) with respect to attenuating fatigue induced performance decrements in competitive athletes are based on the metaanalysis published by Poppendieck et al..[2] Across cooling methods, fatigue protocols, performance measures and timelines the estimated advantage with cooling was 2.4% of initial performance. Depending on the criterion performance higher values are reported for sprint (2.6 %) and lower values for strength (1.8 %). With respect to the cooling method, whole-body cold-water immersion is reported to be most effective (5.1 % of initial performance with an effect size (Hedge's g) of 0.62 [0.45 – 0.80]. Taken together it seems plausible to expect an attenuation of fatigue induced performance decrements around 5% of initial performance for the combination of whole-body CWI and sprint performance. A negative effect seems quite unlikely in this constellation, an expectation which may translate as "2 SD away from 0". Considering mean baseline performances in our subjects (5m acceleration: 0.978 sec, 30 m linear sprint: 4.160 sec), this expectation may be represented by a prior distribution for β_{CWI} of N (0.208, 0.104) sec for 30 m sprint time and of N (0.049, 0.024) sec for 5m acceleration. For comparison, group-based analyses were also conducted with a diffuse prior for β_{CWI} N (0, 100) sec. Priors for intercept and residual variance were N (0, 100) sec and uniform (0, 1000) sec, respectively.

REFERENCES

1. Van de Schoot R, editor. *Small sample size solutions: A guide for applied researchers and practitioners*. 1 ed: Routledge, 2020.
2. Poppendieck W, Faude O, Wegmann M, et al. Cooling and performance recovery of trained athletes: a meta-analytical review. *Int J Sports Physiol Perform* 2013;8:227-42.
3. Hecksteden A, Skorski S, Schwindling S, et al. Blood-Borne Markers of Fatigue in Competitive Athletes - Results from Simulated Training Camps. *PLoS One* 2016;11:e0148810. doi: 10.1371/journal.pone.0148810

4. Senn S. Trying to be precise about vagueness. *Statistics in Medicine* 2007;26:1417-30.
5. Senn S. Transposed conditionals, shrinkage, and direct and indirect unbiasedness. *Epidemiology* 2008;19:652-4; discussion 57-8.
