# Supplementary material for: Dwarfs on the Shoulders of Giants: Bayesian Analysis With Informative Priors in Elite Sports Research and Decision Making
Source: Front Sports Act Living. 2022 Mar 17;4:793603. doi: 10.3389/fspor.2022.793603 (PMC8970347; doi:10.3389/fspor.2022.793603)
Supplement: Supplementary file 2 [file Data_Sheet_1.PDF]

Supplements - Raw data and statistical code

Raw data

Raw data are provided in Excel format (file: Sprint.xls). A file in RData format (Sprint.RData) is available on request. Variable names are explained in the following table.

ID	Subject number<br>(as ordered by posterior β_{exp})
Condition	Experimental condition<br>(control vs. cold-water immersion (CWI))
Code	Code for experimental condition<br>0 = control<br>1 = CWI
Rep	Replication of the cross-over design<br>1 = first cross-over<br>2 = second cross-over (replication)
FivePre	5m acceleration time in seconds before the standardized exercise bout (baseline in this period)
FivePost	5m acceleration time in seconds the day after exercise and CWI or control (follow-up in this period)
ThirtyPre	30 m sprint time in seconds before the standardized exercise bout (baseline in this period)
ThirtyPost	30 m sprint time in seconds the day after exercise and CWI or control (follow-up in this period)
Diff_5_Sec	Pre-Post difference for 5 m acceleration time
Diff_30_Sec	Pre-Post difference in 30 m sprint time

Statistical code

To improve accessibility and enable use as templates for own analyses, the code is densely commented and restricted to the core analyses.

R and stan files are not accepted as supplemental content. Therefor the code is provided as PDF.

Please note that model and prior for the Baysian analyses are specified in a separate Stan file which must be available in the global environment when the R code is run. Please copy the code to a text editor and save as ".stan".

The following combinations of R and Stan files have to be used.

Analysis	Files needed<br>(plus Sprint.RData)
Baysian analysis<br>of 5 m acceleration time	Baysian_Analysis_Five<br>Stan_Five
Baysian analysis<br>of 30 m sprint time	Baysian_Analysis_Thirty<br>Stan_Thirty
Frequentist analysis	Frequentist_Analysis
